# An Update on *Blastocystis*: Possible Mechanisms of *Blastocystis*-Mediated Colorectal Cancer

**DOI:** 10.3390/microorganisms12091924

**Published:** 2024-09-22

**Authors:** Stefania Tocci, Soumita Das, Ibrahim M. Sayed

**Affiliations:** Department of Biomedical & Nutritional Sciences, Zuckerberg College of Health Sciences, University of Massachusetts Lowell, Lowell, MA 01854, USA; stefania_tocci@uml.edu

**Keywords:** *Blastocystis*, CRC, infection-mediated inflammation, microbial diversity

## Abstract

*Blastocystis* is an anaerobic parasite that colonizes the intestinal tract of humans and animals. When it was first discovered, *Blastocystis* was considered to be a normal flora with beneficial effects on human health, such as maintaining gut hemostasis and improving intestinal barrier integrity. Later, with increasing research on *Blastocystis*, reports showed that *Blastocystis* sp. is associated with gastrointestinal disorders, colorectal cancer (CRC), and neurological disorders. The association between *Blastocystis* sp. and CRC has been confirmed in several countries. *Blastocystis* sp. can mediate CRC via similar mechanisms to CRC-associated bacteria, including infection-mediated inflammation, increased oxidative stress, induced gut dysbiosis, and damage to intestinal integrity, leading to a leaky gut. IL-8 is the main inflammatory cytokine released from epithelial cells and can promote CRC development. The causal association of *Blastocystis* sp. with other diseases needs further investigation. In this review, we have provided an update on *Blastocystis* sp. and summarized the debate about the beneficial and harmful effects of this parasite. We have also highlighted the possible mechanisms of *Blastocystis*-mediated CRC.

## 1. Introduction

*Blastocystis* sp. is an anaerobic protist that belongs to the phylum *Stramenopiles* [1]. *Blastocystis* sp. colonizes the guts of humans and several animals, and the parasites are excreted in stool [2]. *Blastocystis* sp. is present in environmental samples such as water, which could pose a risk of infection via the fecal–oral route [3,4,5,6]. *Blastocystis* sp. parasitizes non-human primates [7,8], ungulates [9,10], carnivorous animals [11,12], reptiles [13], rodents [14], and birds [15]. The zoonotic transmission of *Blastocystis* sp. from animals to humans has been documented [16]. A high prevalence of *Blastocystis* sp. (more than 50%) was reported in animal and bird handlers [17,18]. A molecular analysis revealed that the *Blastocystis* sp. isolated from zookeepers and animals in the same area were identical [16]. In addition, human-to-human contact is another source of *Blastocystis* transmission [19].

The prevalence of *Blastocystis* was higher in the stool of CRC patients compared to non-cancer patients, indicating that there is an association between *Blastocystis* and CRC development [20]. There are several methods that can be used to detect *Blastocystis* such as microscopy, the culture method, and molecular assays. Light microscopy is used to detect the morphological forms of the parasites. However, it is not a sensitive method, and the morphology of parasites could be affected by environmental conditions [2]. The culture method is used for parasite cultivation and growth, but there is no selective media for *Blastocystis*, and several other microbes can grow on the same media. In addition, it is time-consuming. Therefore, molecular approaches such as conventional and quantitative PCR are the best methods for the detection of *Blastocystis* [2].

Based on the differences in the small subunit ribosomal rRNA (SSU rRNA) gene sequence, *Blastocystis* sp. isolates include about twenty-eight subtypes [21,22,23,24]. Ten subtypes (ST1-9 and ST 12) are documented as human pathogens [25]. Recent studies have reported other subtypes (ST 18-28), although there is an ongoing debate about whether to include these isolates as new subtypes [21]. ST1, ST2, ST3, and ST4 are highly distributed globally and these isolates were confirmed in human, animal, and environmental samples in the same region [26,27]. ST1 is highly present in the Americas, while ST3 is the most common subtype isolated from humans and animals [28]. The *Blastocystis* subtypes determine the pathogenicity of the protists as pathogens or normal flora [29]. Additionally, several subtypes were also recorded in precancerous tissues (polyps) in infected rats. ST1 and ST3 were highly recorded in the polyps in the colons of infected rats, while ST4 was less abundant (1.9%) [30]. Interestingly, there is an intra-subtype variation that could also affect the pathogenesis of *Blastocystis* [30]. Using molecular approaches (high-resolution melting curve), Hussein and colleagues reported that ST3 could be discriminated into three variants: wild type, heterozygous, and mutant type [30]. However, ST1 and ST4 do not have genetic variations. Wild-type ST3 was detected in 12.9% of the polyps, while the mutant and heterozygous variants were detected less frequently (about 5.5%) [30].

The mechanisms of CRC development have not been studied in depth. In this review, we highlighted the possible mechanisms by which *Blastocystis* could mediate CRC.

## 2. Beneficial and Harmful Effects of *Blastocystis*

### 2.1. Is Blastocystis a Friend?

*Blastocystis* is commonly present in the human intestine as part of the normal flora. About 30% of individuals carry *Blastocystis* sp. without clinical manifestations [31]. *Blastocystis* colonization affects the host’s microbiome composition, leading to a richness of some bacteria sp. (such as *Prevotella*, *Ruminococcus*, and *Faecalibacterium*) and reducing other bacteria such as *Bacteroides* and *Clostridium* [32,33]. Interestingly, Audebert and colleagues showed that *Blastocystis* colonization is linked with a healthy gut rather than with dysbiosis-related gut disorders, cancer, or inflammation [34]. The initial opinion was that the different *Blastocystis* subtypes affect the disease outcome; however, a recent study showed that ST4 (axenized isolate ST4-WR1, isolated from healthy Wistar rat in Singapore) is a beneficial commensal [35]. ST4 increases the production of short-chain fatty acids, regulates T-helper 2 and T-regulatory cells, increases the release of anti-inflammatory IL-10 cytokines, and promotes recovery from DSS-induced colitis [35]. Therefore, *Blastocystis* ST4 improves the stability of gut microbiota [36]. In addition, *Blastocystis* stimulates the intestinal epithelial cells to release antimicrobial peptides, mainly LL-37 [37]. Similarly, Billy and colleagues reported that long-term *Blastocystis* ST3 colonization in rats attenuated the gut inflammation and colitis and enhanced their recovery by affecting the gut ecosystem, reducing inflammatory cytokines (TNF-α and IL-1β), and stimulating IL-17 (IL17re/IL17C) transcripts [38]. The authors explained the previous findings with the fact that the gut of asymptomatic individuals include a high level of *Blastocystis* sp. compared to the gut of patients with gastrointestinal disorders [38]. Likewise, Deng and colleagues reported that ST1 reduced the severity of DSS-induced colitis in mice by activating beneficial bacteria such as *Alloprevotella* and *Akkermansia*, affecting T-cell responses, and increasing the production of short-chain fatty acids [39]. Although ST1 is associated with human diseases, colonization with ST1 could be beneficial for the human gut by positively affecting gut microbiota composition [39].

### 2.2. Is Blastocystis a Foe?

Previous studies have revealed that *Blastocystis* sp. is associated with colorectal cancer (CRC) [20,40], cancers outside the gastrointestinal tract (GIT) [41], gastrointestinal disorders [42], and neurological disorders [43]. Our focus is on CRC and the potential mechanisms *Blastocystis* sp. may initiate or contribute to its progression. *Blastocystis* sp. was abundant in inflammatory bowel disease (IBD) patients, with a percentage of 75% in Crohn’s disease (CD) and 37.1% in ulcerative colitis (UC) patients [42]. Interestingly, a high prevalence of *Blastocystis* sp. was recorded in clinical symptomatic IBD patients, especially CD indicating an association between *Blastocystis* and CD [42]. A recent study showed that the subtypes of *Blastocystis* sp. could affect the outcome of colitis in the DSS-mediated colitis mouse model. ST7 infection increased the severity of colitis in mice, while ST4 reduced the symptoms of colitis in mice [44]. Importantly, IBD is a risk factor for CRC [45], therefore *Blastocystis* can affect CRC development through its effect on colonic inflammation which is a feature of IBD.

#### 2.2.1. *Blastocystis* sp. and CRC

CRC is the second leading cause of cancer-related death in the United States. It has been estimated that about 153,020 individuals have been diagnosed with CRC and a third of them have died from the disease during 2023 [46]. Microbial dysbiosis is one of the risk factors associated with CRC development, and several microbes were identified as potential contributors to CRC initiation and/or progression, such as *Fusobacterium nucleatum*, *Bacteroides fragilis*, *genotoxin-producing E. coli*, *Helicobacter pylori*, and others [47]. Though some are commensals, these microbes can affect CRC development through an infection-mediated inflammation mechanism [47]. Regarding *Blastocystis* sp., several studies have reported a significantly high prevalence of *Blastocystis* sp. in CRC patients compared to a control population [20,48,49,50,51,52]. In one study, the prevalence of *Blastocystis* among CRC patients was 60% which was significantly higher than the prevalence in the cancer-free group (17.3%). ST2 was the main subtype recorded in CRC patients, while ST3 was mainly documented in cancer-free individuals [53]. *Blastocystis* sp. was detected in the feces or colonic washes of CRC patients before and after chemotherapy and/or surgery [20,48,49,50,51,52]. Therefore, screening for *Blastocystis* sp. should be performed during CRC diagnosis and treatment [49].

*Blastocystis* sp. is predominant in CRC patients with higher coloscopy grades; a higher prevalence of protists was documented in the later stages of CRC (grades 3 and 4) [20,54]. A higher prevalence of *Blastocystis* sp. was associated with a high level of inflammatory cells and increased serum level of tumor necrosis factor-α [54]. ST1, ST2, ST3, ST4, ST5, and ST7 were detected in CRC patients from different countries [40,41,55,56,57]. There was no difference in clinical presentations or demographic characteristics between CRC patients infected with different *Blastocystis* subtypes [57].

#### 2.2.2. Possible Mechanisms of *Blastocystis*-Mediated CRC

The mechanisms of *Blastocystis*-mediated CRC are understudied. First, we asked if *Blastocystis* sp. can mediate CRC or is only associated with the changes in the tumor microenvironment that develop during CRC progression. Using CRC animal models, Kumarasamy et al. assessed the effect of *Blastocystis* infection (ST3, isolated from the stool of asymptomatic human) in a rat model challenged with the carcinogen azoxymethane (AOM) [58]. *Blastocystis* infection increased aberrant foci and adenoma in the colons of animals [58]. Co-administration of AOM and *Blastocystis* increased the number of colonic aberrant crypt foci, intensified lesion areas, mucosal layer sloughing, lamina propria inflammation, and increased the adenoma incidence and numbers per colon [58]. Besides, *Blastocystis* ST3 infection increases oxidative stress and the levels of urinary advanced oxidative protein products (AOPP) and hydrogen peroxide [58]. This study confirms that *Blastocystis* infection exacerbates CRC via disruption of the gut epithelium [58]. Using an in vitro colorectal cancer cell line (HCT116), two studies showed that the *Blastocystis* antigen (*Blastocystis hominis* isolated from the stool of symptomatic individuals) promotes HCT116 proliferation by stimulating the nuclear factor kappa light chain enhancer of activated B cells (NF-κB), Cathepsin B, and proinflammatory cytokines that stimulated cell proliferation, invasion, and metastasis [59,60]. These studies support the hypothesis that *Blastocystis* infection participates in the development of CRC.

**Figure 1:**
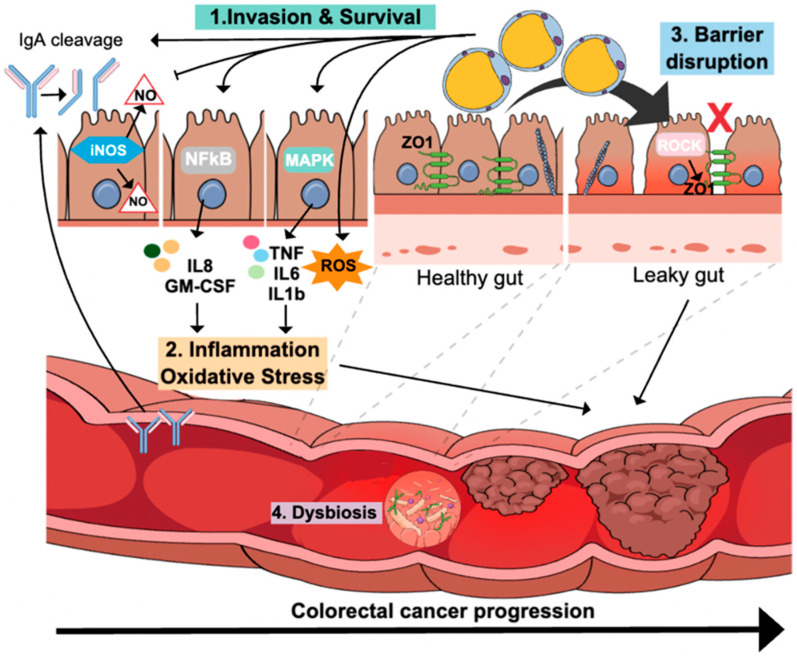
Possible mechanisms through which *Blastocystis* can promote CRC. (1) Invasion of and survival in the host by targeting the immune responses through the degradation of IgA and suppression of antimicrobial products, iNOS. (2) *Blastocystis* infection induces an infection-mediated inflammation by stimulating the release of inflammatory cytokines, mainly IL-8, TNF-α, IL-6, and IL-1β, and oxidative stress that promotes gut leakiness and stimulates the oncogenesis pathways. (3) *Blastocystis* disrupts the intestinal barrier inducing a leaky gut by targeting ZO-1 protein. (4) *Blastocystis* infection causes gut microbial dysbiosis.

In Figure 1, we summarized the potential mechanisms of *Blastocystis*-mediated CRC. *Blastocystis* sp. invades and escapes the immune system to survive in the gut. *Blastocystis hominis* isolate B exhibits immunomodulatory effects, including the degradation of host Immunoglobulin IgA [61]. Additionally, *Blastocystis* infection (ST7) suppresses intestinal epithelial inducible nitric oxide synthase (iNOS) to inhibit Nitric Oxide (NO) production, which is considered an important antimicrobial host defense [62]. *Blastocystis* infection can mediate CRC through an infection-mediated inflammation mechanism similar to *Fusobacterium*-mediated CRC and *H.plyori*-mediated gastric cancer as described in our previous studies [63,64]. *Blastocystis hominis* and *Blastocystis* ratti WR1 stimulate the release of inflammatory cytokines from intestinal epithelial cells, especially IL-8, by affecting NF-κB [65,66]. IL-8 interacts with the CXC chemokine receptor and activates the signaling pathways, including protein kinase B (Akt), mitogen-activated protein kinase (MAPK), extracellular signal-regulated kinase 1/2 (ERK1/2), signal transducer and activator of transcription 3 (STAT3), and SNAIL [67,68]. IL-8 stimulates stem cell activity, cellular proliferation, epithelial–mesenchymal transition (EMT), neutrophil stimulation, angiogenesis, and the migration of CRC cells [67]. In our previous study, we showed that IL-8 is the main cytokine released from colonic epithelial cells infected with CRC-associated bacteria that can promote CRC progression [63]. Similarly, *Blastocystis* can promote CRC through the induction of IL-8. Besides, *Blastocystis* infection (ST7 and ST4) upregulates the release of other proinflammatory cytokines such as IL-1β, TNF-α, and IL-6 [60,69], which have been shown to promote CRC progression through various signaling pathways [70,71,72,73]. A recent study showed that *Blastocystis* ST7 infection increased pro-inflammatory Th17 and decreased anti-inflammatory Treg cells by affecting tryptophan metabolite indole-3-acetaldehyde (I3AA) [74]. I3AA mediated by *Blastocystis* ST7 increased the T-cell responses against self-microbiome and mimics aryl hydrocarbon receptor (AhR) inhibitor, suggesting a possible mechanism for the induction of inflammatory responses in the gut [74].

The trans-epithelial permeability of intestinal cells is regulated by tight junctions (TJs) and is critical in creating a protective barrier against microbial pathogens. Disruption of epithelial TJs leads to increased intestinal permeability, resulting in a leaky gut, which is a risk factor for CRC [75]. Zonula occludens (ZOs) proteins play a role in maintaining the epithelial TJs and ZO-1 is a tumor-suppressive protein [76]. Reduced expression of ZO-1 causes increased intestinal permeability and increased proliferation of CRC epithelial cells [75,76]. Several studies have shown that a *Blastocystis* infection (mainly ST7) increases intestinal epithelial permeability, which could be attributed to the rearrangement of F actin filaments and the destruction of epithelial TJs [77,78,79]. Importantly, *Blastocystis* targets ZO-1 expression and organization via its effect on caspase 3 and 9 enzymes which seems to be subtype dependable [78]. ST7 infection causes a reduction in the activity of caspase 3 and caspase 9, but not caspase 8 after 6 and 12 h of infection. On the other hand, ST4 infection did not affect the activity of the previous caspase enzymes [78]. Another study reported that *Blastocystis* cysteine proteases cause the disruption of the intestinal barrier via ROCK-dependent mechanisms that target cytoskeletal F-actin and ZO-1 [80].

Moreover, Nourrisson et al. demonstrated that the cysteine protease produced by *Blastocystis* ST7, Cathepsin B, is linked to increased Caco-2 cell monolayer permeability [79]. Cathepsin B enhances colon carcinogenesis, metastasis, and cell invasion [81]. Several other studies supported this hypothesis and showed that *Blastocystis* antigen induces cathepsin B production, which affects p53 and NF-κB, increasing cancer cell growth and proliferation [59,60,82].

It seems that *Blastocystis* infection could affect the following pathways: infection-mediated inflammation, increased oxidative stress, and disruption of epithelial TJs through targeting epithelial ZO-1 and producing cathepsin B. The previous pathways are risk factors for CRC development; however, the direct cause of CRC by *Blastocystis* infection needs further investigation.

#### 2.2.3. *Blastocystis* sp. and Other Cancers

*Blastocystis* sp. was also recorded in patients with cancers outside the gastrointestinal tract (GIT). *Blastocystis* sp. was detected in patients with hematological malignancies (such as leukemia, lymphoma, and multiple myeloma), bladder cancer, breast cancer, lung cancer, pancreatic cancer, basal cell carcinoma, laryngeal cancer, renal cell carcinoma, and prostate cancer [4,40,41,53,83]. Labania et al. reported a lower prevalence of *Blastocystis* sp. in patients with cancers outside the GIT compared to CRC patients [53]. However, other studies reported the opposite findings and the prevalence of *Blastocystis* sp. was higher in patients diagnosed with lung and breast cancer than CRC patients [40,41]. Several subtypes were found in cancer patients, such as ST1, ST2, ST3, ST4, and ST7 [40,41,53]. An important aspect is the high frequency of *Blastocystis* in cancer patients who undergo numerous chemotherapy cycles [53,84], suggesting that *Blastocystis* is an opportunistic pathogen in patients with compromised immune systems. Whether *Blastocystis* sp. is only an opportunistic pathogen in this category or if it can mediate cancer development by specific pathways needs further investigation. Likewise, *Blastocystis* causes opportunistic infections in other immunocompromised populations, such as HIV/AIDS patients and transplant recipients [85,86].

#### 2.2.4. *Blastocystis* sp. and Other Gastrointestinal Disorders

*Blastocystis* sp. causes gastrointestinal disorders such as inflammatory bowel syndrome (IBS) and IBD. The link between *Blastocystis* infection and intestinal disorders dates back to the late 20th century when a high prevalence of *Blastocystis* in individuals with IBS symptoms was reported [87]. IBS/IBD are multifactorial disorders influenced by genetic, environmental, and microbiological factors, and studies conducted in different geographic areas may contribute to the discrepancies in the findings about *Blastocystis* in IBS/IBD development. *Blastocystis* was detected in IBS patients in India (33.3%) [88], Iran (19.7%) [89], Indonesia (36.5%) [90], and Thailand (16.7%) [91]. However, the prevalence was significantly higher in IBS patients compared to controls in some studies [88,90], and not different in other studies [89,91,92], raising questions about the ability of a *Blastocystis* infection to cause IBS [92]. Similarly, the prevalence of *Blastocystis* sp. was not significantly different in IBD patients [92,93]; however, ulcerative colitis patients had a significantly higher prevalence than the controls [92]. Using an in vivo DSS-induced colitis model, Yason and colleagues showed that *Blastocystis* ST7 worsens colitis symptoms due to gut dysbiosis, particularly by suppressing beneficial bacteria such as *Bifidobacterium* and *Lactobacillus* [94]. Later, another study reported that *Blastocystis* subtypes could affect the outcome of colitis in a DSS-induced mouse model [44]. *Blastocystis* ST4 reduces the severity of colitis due to an increasing number of beneficial bacteria producing short-chain fatty acids and increasing the number of T-regulatory cells and T-cells that release anti-inflammatory cytokines [44]. On the other hand, *Blastocystis* ST7 increases the severity of colitis due to increasing the number of harmful bacteria and increasing the number of -T-cells that release inflammatory cytokines such as IL-17 and TNF-α. In line with this, though *Blastocystis* ST1 is prevalent among human diseases, a recent study showed that ST1 ameliorated the severity of DSS-induced colitis by affecting gut microbe and gut immune responses [39]. The discrepancies in the effect of *Blastocystis* on IBS or IBD could be related to the subtype/strains used, experimental approaches, differences in the gut microbiota among animals, and differences between the effect of infection on human and animal models. We believe that the link between *Blastocystis* and IBS and IBD needs more research. Since IBS and IBD are affected by the gut microbiome, the role of *Blastocystis* sp. on microbial diversity and microbial ecosystem is debated.

#### 2.2.5. *Blastocystis* sp. and Neurological Disorders

*Blastocystis* sp. could be linked directly or indirectly to neurological disorders because of its effect on tryptophan production in the gut [95,96]. Leonardi and colleagues reported that *Blastocystis* is a possible producer of tryptophan in the gut via the effect of the tryptophanase gene (BhTnaA) [96]. In addition, *Blastocystis* sp. alters the gut microbiome diversity, which is another source of tryptophan in the gut [95]. Tryptophan and its metabolites such as indole, serotonin, and Kynurenine are linked to depression [97]. Another study performed by Mayneris-Perxachset et al. showed that *Blastocystis* has been associated with impairment of cognitive functions and deficit in executive functions, along with altered gut microbial composition in patients and human-microbiota-transplanted mice, supporting the recent growing hypothesis of a possible gut–brain axis [43].

## 3. Discussion and Future Exploration

**Figure 2:**
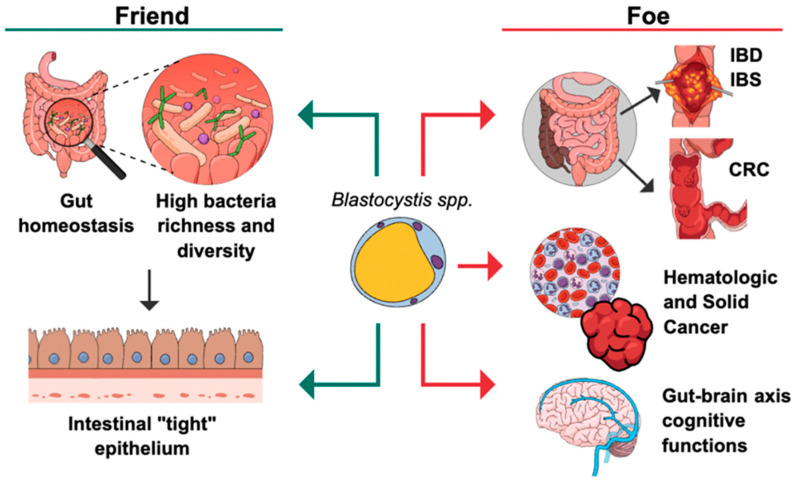
Summary of beneficial and harmful effects of *Blastocystis*. *Blastocystis* can part of the normal flora and maintain the gut hemostasis by increasing the beneficial bacteria in gut. Also, it can increase intestinal TJs and gut barrier integrity. On the other hand, several diseases were reported in association with *Blastocystis* (foe) such as CRC, cancer outside the gut, gastrointestinal disorders (IBD and IBS), and neurological disorders.

The association of *Blastocystis* sp. with human disease is still up for debate (summarized in Table 1). Initially, researchers considered *Blastocystis* sp. to be a normal flora (friend); however, with increasing research in this field, it becomes clear that *Blastocystis* sp. is linked directly or indirectly to human diseases (foe). In Figure 2, we summarize the beneficial and harmful effects of *Blastocystis* sp. However, certain aspects remain unclear. While *Blastocystis* sp. alters the gut microbiome, there is ongoing debate about whether this alteration favors an increase in beneficial bacteria or an increase in harmful ones. One study showed that the subtype of *Blastocystis* can influence the preferences of bacteria; ST4 increases beneficial bacteria, while ST7 increases harmful ones [44]. However, *Blastocystis* ST4 was documented in IBS patients, CRC patients, and other immunocompromised patients [4]. Similarly, though ST3 is one of the common subtypes recorded in different patient categories, a recent study showed that long-term colonization of ST3 attenuates colitis and speeds up recovery by altering the gut microbiome [38]. It would be beneficial to classify *Blastocystis* strains belonging to the same subtype, and it is possible that a difference in pathogenicity between the isolates within the same subtype exists. Deep next-generation sequencing, proteomics, and other advanced approaches could help in assessing these points. This review discussed the reports that link *Blastocystis* sp. to CRC development. Although prevalence data from human studies, in vivo animal models, and in vitro research support this association, there are still missing data regarding the signaling pathways that *Blastocystis* activates during CRC such as stemness pathways and epithelial–mesenchymal transition. Future studies including transcriptomic and proteomic analyses of *Blastocystis*-infected CRC patients could help us to understand the mechanisms and signaling pathways that the protist stimulates to affect the cancer’s progress. Also, most of the reported studies identified *Blastocystis* in patients’ stools. The inclusion of patients’ colon samples, especially from the areas in which the adenoma or cancer has developed could help to assess the risk of *Blastocystis*-mediated CRC. Treatments for *Blastocystis* infection include metronidazole, nitazoxanide, and trimethoprim–sulfamethoxazole [98]. One study showed that metronidazole treatment could reduce cancer growth, and cell proliferation in mice infected with *Fusobacterium* [99]; however, to our knowledge, no study assesses the effect of anti-*Blastocystis* therapies on CRC development. It is possible that metronidazole could be beneficial in CRC cases, and future studies should confirm this.

Moreover, the link between *Blastocystis* sp. with IBS and IBD needs further investigation due to conflicting findings in both human studies and in vivo animal model, with more focus on the pathophysiology and mechanisms caused by *Blastocystis* sp. in these diseases.

Most research focuses on the detection and characterization of *Blastocystis* in human, animal, or environmental samples. However, in vivo and in vitro studies are limited. Therefore, our knowledge of the pathogenesis of *Blastocystis* sp. in different diseases is not complete. Also, the lack of a suitable cell model system that mimics the human microenvironment is another challenge. Recently, our group developed stem-cell-based 3D organoid models from human and CRC-murine animal models to study the pathogenesis of CRC-associated pathogens, [63,64] offering a platform to study the pathogenesis of *Blastocystis* in the CRC. Additionally, our group developed 3D organoids from IBD patients [104], which can similarly provide a model to explore the association of *Blastocystis* infection and IBD.

The impact of the coinfection between two microbes (bacteria, fungi, or parasites) on disease outcome is another hot topic research. A study performed by Dejea et al. showed that patients with familial adenomatous polyposis (FAP) harbor two carcinogenic bacteria in their colons (*B. fragilis* and colibactin-producing *E. coli*), and those patients develop polyps early in life [105]. Likewise, the impact of *Blastocystis* sp. and other tumorigenic bacteria and IBD-associated bacteria should be studied to determine the outcomes of *Blastocystis* coinfection with other microbes in disease pathogenesis.

Moreover, since the subtypes affect the pathogenicity and disease outcomes. Future studies should focus on the specific subtype/strain and explore its effects on host health at different experimental treatment conditions. Characterization of specific subtypes/strain is crucial to identify the exact beneficial or harmful effects of these isolates.

## 4. Conclusions

With increasing research on *Blastocystis* sp., it becomes clear that not all subtypes are friends to humans. Some subtypes may be linked to diseases such as CRC. *Blastocystis* sp. infections can initiate and promote CRC progression through inflammation, inducing leaky gut, altering the gut microbial diversity, and stimulating host DNA damage.

## Figures and Tables

**Table 1:** Harmful and beneficial effects of common *Blastocystis* subtypes that are detected in humans.

*Blastocystis* sp. Subtype	Harmful Effects (Detected in)			Beneficial Effects
	Colorectal Cancer (CRC)	Other Gastrointestinal Disorders	Cancer Outside the GIT	
ST1	Yes [30,40,41,48,56]	Yes, IBS [88,90]	Yes, such as Lymphoma, pancreatic cancer, basal cell carcinoma, multiple myeloma, prostate cancer, laryngeal cancer, liver cancer, and lung cancer [40,41,48,56]	ST1 ameliorates DSS-induced colitis, promotes beneficial microbiota, and induces accumulation of Th2 and Treg cells [39]
ST2	Yes [53,57]	Yes, IBS [100]	Yes, such as Lung cancer, breast cancers, and cancer outside GIT [41,48,57]	-
ST3	Yes [40,41,52,57]	Yes, IBS [88,90]	Yes, such as Lymphoma, Bladder cancer, Basal cell carcinoma, endometrial cancer, beast and stomach cancer [40,41,57]	Long-term colonization of ST3 attenuates colitis and inflammation in rat model and speeds up recovery by altering the gut microbiome [38].
ST4	Yes [40,48]	Yes, IBS [101] In one study, no difference between ST4 in the prevalence of IBS patients and healthy controls [102]	Yes, such as Lymphoma, Bladder cancer, pancreatic cancer, stomach cancer, lung cancer, and others [40,48]	Yes, prior colonization provides protection from DSS-induced colitis by enrichment of beneficial microbiota and short chain fatty acids [35,44] Also affects intestinal microbiota stability [36]
ST7	Yes [20]	Yes. Affects diarrhea patients by decreasing bacteria diversity [103]		

## Data Availability

All data generated are present in the manuscript. For further inquiries, please contact the corresponding authors.
